# Breastfeeding and cardiometabolic risk factors in adulthood: results from the Pelotas (Brazil) birth cohort, 1982

**DOI:** 10.1590/0102-311XEN044224

**Published:** 2025-02-24

**Authors:** Natália Peixoto Lima, Janaína Vieira dos Santos Motta, Bernardo Lessa Horta

**Affiliations:** 1 Programa de Pós-graduação em Epidemiologia, Universidade Federal de Pelotas, Pelotas, Brasil.; 2 Departamento de Medicina Social, Universidade Federal de Pelotas, Pelotas, Brasil.

**Keywords:** Breast Feeding, Heart Disease Risk Factors, Birth Cohort, Aleitamento Materno, Fatores de Risco para Doenças Cardíacas, Coorte de Nascimentos, Lactancia Materna, Factores de Riesgo de Enfermedad Cardiaca, Cohorte de Nacimientos

## Abstract

Using data from a birth cohort study, we evaluated the long-term association between breastfeeding and metabolic cardiovascular risk factors at 30 years old. In 1982, the 5,914 live births in maternity hospitals in Pelotas, Rio Grande do Sul State, Brazil, were examined and the mothers were interviewed. Since then, these participants have been prospectively followed. The cohort members were interviewed at 30 years old and information on blood pressure, carotid intima-media thickness, pulse wave velocity, random blood glucose, and blood lipids were obtained. Simple and multiple linear regressions were used. In the crude and adjusted analyses, duration of breastfeeding did not show any clear association with mean arterial pressure, carotid intima-media thickness, and pulse wave velocity. Likewise, total and non-HDL cholesterol and blood glucose were not related to infant feeding. Regarding HDL cholesterol, it was positively related to predominant breastfeeding duration in the crude analysis, but the association disappeared after controlling for confounding variables. Concisely, our findings suggest that there is no association between duration of breastfeeding and cardiometabolic risk factors in adulthood. Despite that, promotion and support of breastfeeding must be reinforced due to its well-known benefits, such as reduction of infant and child mortality and human capital development.

## Abstract (pt)

Utilizando dados de um estudo de coorte de nascimento, foi avaliada a associação a longo prazo entre amamentação e fatores de risco cardiovascular metabólicos aos 30 anos de idade. Em 1982, foram examinados 5.914 bebês nascidos vivos em maternidades de Pelotas, Rio Grande do Sul, Brasil, e suas mães foram entrevistadas. Desde então, esses indivíduos têm sido continuamente acompanhados. Aos 30 anos de idade, os membros da coorte foram entrevistados e foram obtidas informações sobre pressão arterial, espessura da íntima-média da carótida, velocidade da onda de pulso, glicemia aleatória e lipídios sanguíneos. Foi utilizada regressão linear simples e múltipla. Nas análises bruta e ajustada, a duração da amamentação não apresentou nenhuma associação clara com a pressão arterial média, a espessura da íntima-média da carótida e a velocidade da onda de pulso. Da mesma forma, o colesterol total, o colesterol não-HDL e a glicemia não apresentaram relação com a alimentação infantil. Em relação ao colesterol HDL, houve uma associação positiva com a duração da amamentação predominante na análise bruta, mas a associação não persistiu após o controle das variáveis de confusão. Em suma, os nossos achados sugerem que não há associação entre a duração da amamentação e os fatores de risco cardiometabólicos na idade adulta. Apesar disso, a promoção e o apoio à amamentação devem ser reforçados devido aos seus benefícios bem conhecidos, como a redução da mortalidade infantil e o desenvolvimento do capital humano.

## Abstract (es)

Utilizando datos de un estudio de cohorte de nacimiento, se evaluó la asociación a largo plazo entre la lactancia materna y los factores de riesgo cardiovascular metabólicos a los 30 años de edad. En 1982, se examinaron 5.914 bebés nacidos vivos en hospitales de maternidad en Pelotas, Rio Grande do Sul, Brasil, y se entrevistó a sus madres. Desde entonces, estos individuos pasaron por un seguimiento continuo. A los 30 años de edad, se entrevistó a los miembros de la cohorte y se obtuvo información sobre la presión arterial, el grosor íntima-media carotídeo, la velocidad de la onda del pulso, la glucosa en sangre aleatoria y los lípidos en sangre. Se utilizó regresión lineal simple y múltiple. En los análisis bruto y ajustado, la duración de la lactancia materna no mostró una asociación clara con la presión arterial media, el grosor íntima-media carotídeo y la velocidad de la onda del pulso. Del mismo modo, el colesterol total, el colesterol no HDL y la glucosa en sangre no estaban relacionados con la nutrición infantil. Con respecto al colesterol HDL, hubo una asociación positiva con la duración de la lactancia materna predominante en el análisis bruto, pero la asociación no persistió tras controladas las variables de confusión. Los hallazgos indican que no existe una asociación entre la duración de la lactancia materna y los factores de riesgo cardiometabólico en la edad adulta. A pesar de esto, la promoción y el apoyo a la lactancia materna deben fortalecerse debido a sus beneficios ya conocidos, como la reducción de la mortalidad infantil y el desarrollo del capital humano.

## Introduction

In the last decades, noncommunicable diseases (NCDs) have emerged as the leading cause of the global burden of disease ^1^. Annually, NCDs result in approximately 17 million premature deaths worldwide, with cardiovascular diseases being the primary cause of mortality globally ^2^. Factors such as high blood pressure, plasma glucose levels, and LDL cholesterol were among the top 10 leading risk factors for disability-adjusted life years in 2021, with high blood pressure standing as the main contributor ^1^.

Breastfeeding has clear short-term (reducing morbidity and mortality from infectious diseases ^3^) and long-term benefits. In the long-term, breastfeeding would be positively associated with human capital ^4^ ^,^ ^5^ ^,^ ^6^ and would decrease the risk of NCDs. A recently published meta-analysis reported that breastfeeding would protect against overweight/obesity (pooled odds ratio - pooled OR = 0.85 and 95% confidence interval - 95%CI: 0.77; 0.93) ^7^. Moreover, breastfeeding would also decrease the odds of type 2 diabetes (pooled OR = 0.67, 95%CI: 0.56; 0.80) ^8^. Furthermore, Lewandowski et al. ^9^ observed that preterm subjects who had been exclusively breastfed had increased left and right ventricular end-diastolic volume index, in relation to those who had not been breastfed. The plausibility of the association between breastfeeding and overweight/obesity is emphasized by the finding that breastfeeding moderates the association of the fat mass and obesity-associated (FTO) genotype with obesity ^10^.

On the other hand, most studies on the long-term consequences of breastfeeding are observational and have been conducted in high-income countries ^11^, in which socioeconomic status is positively associated with duration of breastfeeding ^6^. Therefore, the observed associations could be due to residual confounding by socioeconomic status. In the 1982 Pelotas (Brazil) birth cohort, breastfeeding duration was not related to socioeconomic status ^12^. Consequently, studies using the cohort data provide evidence that are not susceptible to residual confounding.

This study aimed to assess the long-term association of duration of breastfeeding and metabolic cardiovascular risk factors at 30 years old (mean arterial pressure, carotid intima-media thickness, pulse wave velocity, glycemia, and total, HDL, and non-HDL cholesterol), using data from a birth cohort study conducted in Pelotas, a southern Brazilian city. The study hypothesis was: duration of breastfeeding was positively associated with HDL and inversely associated with the other cardiovascular risk factors evaluated.

## Methods

This study used data from the 1982 Pelotas (Brazil) birth cohort. In 1982, the maternity hospitals in Pelotas were visited daily and all births recorded. Those live births (n = 5,914) whose family lived in the urban area of the city were examined and the mothers were interviewed. Initially, the cohort aimed to investigate perinatal, infant, and early childhood morbidity and mortality ^13^. Over time, the study expanded to include follow-ups into later stages of life, with cohort participants undergoing multiple assessments ^13^ ^,^ ^14^. In 2012-2013, we tried to locate all cohort members and invited the subjects to attend the research clinic to be interviewed and examined ^15^.

The study protocol of the 1982 Pelotas cohort was approved by the Research Ethics Committee of the Faculty of Medicine of the Federal University of Pelotas (protocol number: Of. 16/12). Written informed consent was obtained from all participants.

### Assessment of breastfeeding

Information on duration of breastfeeding and age at introduction of complementary feeding was gathered in the 12, 24 and 48 months of the cohort visits, and the earliest information on the age at which breastfeeding completely stopped was used. Duration of predominant breastfeeding was defined as the age when foods other than breast milk, water and tea were introduced. Considering that exclusive breastfeeding was an unusual practice at that time and had a short duration in the sample, it was included in the predominant breastfeeding group.

### Outcomes assessment

At 30 years, metabolic cardiovascular risk factors were evaluated. Blood pressure was assessed twice on the left arm, with a specific cuff for individuals with obesity, using a digital sphygmomanometer (Omron HEM 705CPINT; https://www.omron.com/). The measurement was made at the beginning and the end of the anthropometric evaluation, and the mean of these measures was used in the analyses. For participants who were taking antihypertensive drugs, assuming a linear blood pressure reduction due to antihypertensive treatment, 8 and 5mmHg were added to the values of systolic and diastolic blood pressure, respectively, for each additional drug ^16^ ^,^ ^17^. Afterwards, mean arterial pressure was computed by adding double of the diastolic pressure to the systolic number and then dividing that value by three. Carotid intima-media thickness was assessed at the posterior wall of right and left common carotid in longitudinal planes using a Toshiba Xario ultrasound (https://www.toshiba.com/tic/industries-served/medical). A 10mm-long section of the common carotid artery was imaged proximal to carotid bulb ^18^. The Carotid Analyzer for Research (Medical Imaging Applications, MIA-LLC; https://mia-llc.com/) automatically estimated the mean value of 90 measurements (frames) taken in the 10mm long section studied. Carotid-femoral pulse wave velocity was measured twice using a portable ultrasound, Sphygmocor (Atcor Medical; https://atcormedical.com/) in the supine position. The distance of pulse wave transit was measured with a flexible tape as the distance from suprasternal notch to femoral and carotid application point of the tonometer. Pulse wave velocity was estimated as the distance between the measurement sites divided by transit time delay between femoral and carotid pulse wave. The mean of the measurements was used in the analysis. Random blood glucose and total and HDL cholesterol were obtained using an automatic enzymatic colorimetric method in a chemistry analyzer (BS-380, Shenzhen Mindray Bio-Medical Electronics Co.; https://www.mindray.com/en). Non-HDL cholesterol was computed by subtracting HDL from total cholesterol.

### Statistical analysis

Statistical analyses were performed using Stata, version 15 (https://www.stata.com/). Means were compared using analysis of variance (ANOVA), and the chi-square test was used to compare proportions. Simple and multiple linear regressions were used to evaluate the association between breastfeeding and cardiometabolic outcomes. In the linear regression, the normality of residuals and homoscedasticity were graphically tested. Multivariable models were adjusted for the following confounding factors collected at the perinatal study, informed by previous findings in the literature and theoretical knowledge: sex; family income (≤ 1; 1.1-3; 3.1-6; 6.1-10; > 10 minimum wages); maternal schooling (0-4; 5-8; 9-11; ≥ 12 years); maternal smoking in the pregnancy (yes; no); maternal hypertension or diabetes during pregnancy; maternal age; mode of delivery (vaginal; cesarean), maternal body mass index (BMI) before pregnancy; gestational age, and birthweight -measured soon after childbirth by hospital staff. Furthermore, the models were controlled for household asset index at 24 months, calculated via factor analysis of a list of household durable goods, and European genomic ancestry proportion, based on the analysis of approximately 370,000 single nucleotide polymorphisms (SNPs) accessible for samples of the Pelotas cohort, the HapMap Project, the Human Genome Diversity Project ^19^. For mean arterial pressure, analyses were also adjusted for use of oral contraceptive and height at 30 years, whereas for glycemia, time since last meal was also included in the multivariable model. Significance level was set at 0.05. For ordinal data, both heterogeneity and linear trend were tested, showing the one with the lowest p-value.

## Results

In 2012-2013, we managed to interview 3,701 participants, averaging 30.2 years old, which added to the 325 deaths identified among the cohort members, represented a follow-up rate of 68.1%. Participants were slightly more likely to be female and from intermediate socioeconomic groups compared to the original cohort; although these differences were statistically significant, they were small between the groups, being at most 6.5 percentage points. Regarding breastfeeding duration, there was no significant difference in the follow-up (Table 1).

**Table 1:** Proportion of participants from the original 1982 included in the analysis, according to selected characteristics. Pelotas (Brazil) birth cohort, 2012.

Variables	Original cohort	Included	p-value
	n	n (%) *	
Sex			
Male	3,037	1,718 (60.4)	< 0.001
Female	2,876	1,812 (65.9)	
Family income at birth			
1st tertile	1,963	1,109 (61.7)	0.003
2nd tertile	1,979	1,249 (66.2)	
3rd tertile	1,972	1,172 (61,5)	
Maternal schooling (years)			0.004
0-4	1,960	1,133 (62.3)	
5-8	2,454	1,522 (65.6)	
9-11	654	385 (61.5)	
≥ 12	839	486 (59.1)	
Maternal smoking			0.434
No	3,811	2,303 (63.5)	
Yes	2,103	1,227 (62.5)	
Gestational age (weeks)			0.593
< 37	294	160 (66.1)	
37-39	2,167	1,305 (62.7)	
≥ 40	3,453	2,065 (63.1)	
Type of delivery			0.813
Vaginal	4,282	2,551 (63.0)	
Cesarean	1,632	979 (63.4)	
Birthweight (g)			0.188
< 2,500	534	249 (59.9)	
2,500-2,999	1,393	839 (63.8)	
3,000-3,499	2,220	1,334 (62.2)	
≥ 3,500	1,762	1,107 (64.7)	
Breastfeeding (months)			0.157
< 1	1,171	744 (66.2)	
1 to < 3	1,405	908 (66.7)	
3 to < 6	1,212	819 (69.8)	
6 to < 9	497	335 (69.3)	
9 to < 12	209	138 (67.6)	
≥ 12	838	584 (70.9)	

Note: the definition of family income in tertiles is provided in the Supplementary Material (https://cadernos.ensp.fiocruz.br/static//arquivo/supl-e00044224_6433.pdf).

* The 325 participants who were known to have died were not included in the denominators.

Table 2 shows that about seven out of 10 participants had a family income at birth ≤ 3 minimum wages. Maternal schooling was heterogeneous, whereas about one of every three mothers had ≤ 4 years of schooling, 13.8% had completed middle school (≥ 12 years of schooling). Regarding breastfeeding, 21.1% of the participants were breastfed for less than one month and 30% breastfed for six or more months. Duration of predominant breastfeeding was short, and only about four out of 10 participants were predominantly breastfed for three months or more. At 30 years, 57.7% of the participants were overweight, mean arterial pressure was 90.8mmHg, and mean total cholesterol was 191.3mg/dL.

**Table 2:** Main characteristics of the studied population (n = 3,530). 1982 Pelotas (Brazil) birth cohort, 2012.

Variables	n (%)
Birth	
Sex	
Male	1,718 (48.7)
Female	1,812 (51.3)
Family income at birth (minimum wages)	
≤ 1	699 (19.9)
1.1-3	1,742 (49.5)
3.1-6	684 (19.5)
6.1-10	206 (5.9)
> 10	183 (5.2)
Maternal schooling (years)	
0-4	1,133 (32.1)
5-8	1,522 (43.2)
9-11	385 (10.9)
≥ 12	486 (13.8)
Childhood	
Breastfeeding (months)	
< 1	744 (21.1)
1 to < 3	908 (25.7)
3 to < 6	819 (23.2)
6 to < 9	335 (9.5)
9 to < 12	138 (3.9)
≥ 12	584 (16.6)
Predominant breastfeeding (months)	
< 1	906 (26.5)
1 to < 2	467 (13.7)
2 to < 3	696 (20.4)
3 to < 4	921 (26.9)
≥ 4	427 (12.5)
30 years of age	
Prevalence of overweight (≥ 25.0kg/m^2^)	1,974 (57.7)
Systolic blood pressure (mmHg) [mean (SD)]	121.4 (14.1)
Diastolic blood pressure (mmHg) [mean (SD)]	75.5 (9.5)
Mean arterial pressure (mmHg) [mean (SD)]	90.8 (10.4)
Intima-media thickness (µm) [mean (SD)]	582.3 (18.5)
Pulse wave velocity (m/s) [mean (SD)]	6.4 (1.1)
Glycemia (mg/dL) [mean (SD)]	89.5 (26.3)
Total cholesterol (mg/dL) [mean (SD)]	191.3 (38.2)
HDL (mg/dL) [mean (SD)]	58.7 (13.9)
Non-HDL (mg/dL) [mean (SD)]	132.6 (36.8)

SD: standard deviation.

Table 3 shows that family income at birth was not associated with the prevalence of breastfeeding in the first six months of life. Maternal age, birthweight, and maternal BMI before pregnancy were positively associated with the prevalence of breastfeeding in the first six months. Nonetheless, offspring of mothers who smoked in the pregnancy and those born by cesarean section were less likely of being breastfed in the first six months of life. Mean arterial pressure was positively associated with maternal BMI before pregnancy and hypertension or diabetes during pregnancy. Moreover, carotid intima-media thickness was lower among females, inversely associated with socioeconomic status and positively related to maternal BMI and birthweight, whereas pulse wave velocity was positively associated with maternal BMI before pregnancy. Table 4 shows that glycemia, total and non-HDL cholesterol were lower among females. Blood glucose was lower among participants whose mother did not smoke during pregnancy. Total and HDL cholesterol were directly associated with socioeconomic status at birth and were lower in those with low birthweight.

Table 5 shows that mean arterial pressure, carotid intima-media thickness, and pulse wave velocity did not show any clear association with infant feeding in the crude and adjusted analyses. Table 6 shows that HDL cholesterol was positively related to predominant breastfeeding in the crude analysis, but the association disappeared after controlling for confounding variables. On the other hand, total and non-HDL cholesterol and random blood glucose were not related to infant feeding.

**Table 3:** Breastfeeding in childhood and mean arterial pressure, carotid intima-media thickness and pulse wave velocity at 30 years of age according to confounding variables (n = 3,530). 1982 Pelotas (Brazil) birth cohort, 2012.

Variables	Breastfeeding in the first 6 months	Mean arterial pressure (mmHg)	Carotid intima-media thickness (µm)	Pulse wave velocity (m/s)
%	Mean (95%CI)	Mean (95%CI)	Mean (95%CI)
Sex	p = 0.96 *	p < 0.01 *	p < 0.01 *	p = 0.07 *
Male	29.9	94.2 (93.7; 94.7)	585.3 (584.3; 586.3)	6.47 (6.4; 6.6)
Female	30.0	87.6 (87.1; 88.1)	579.3 (578.6; 580.1)	6.37 (6.3; 6.4)
Family income at birth	p = 0.08 *	p = 0.74 *	p = 0.04 **	p = 0.33 **
1st tertile	32.3	90.8 (90.1; 91.4)	583.1 (581.8; 584.4)	6.47 (6.4; 6.6)
2nd tertile	28.1	91.0 (90.4; 91.6)	582.4 (581.3; 583.5)	6.40 (6.3; 6.5)
3rd tertile	29.7	90.7 (90.1; 91.3)	581.4 (580.5; 582.4)	6.40 (6.3; 6.5)
Maternal prepregnancy BMI	p = 0.04 **	p = 0.01 **	p < 0.01 **	p = 0.04 **
Underweight	23.6	91.2 (89.9; 92.5)	580.2 (578.1; 582.2)	6.43 (6.2; 6.6)
Normal	30.0	90.4 (89.9; 90.8)	581.6 (580.8; 582.4)	6.37 (6.3; 6.4)
Overweight	32.0	91.4 (90.5; 92.3)	583.2 (581.6; 584.8)	6.55 (6.4; 6.7)
Obese	32.2	93.0 (91.3; 94.8)	588.5 (583.6; 593.3)	6.55 (6.2; 6.9)
Maternal smoking	p < 0.01 *	p = 0.84 *	p = 0.23 *	p = 0.36 *
No	33.0	90.8 (90.4; 91.3)	582.0 (581.2; 582.8)	6.40 (6.3; 6.5)
Yes	24.3	90.8 (90.2; 91.3)	582.8 (581.6; 584.1)	6.46 (6.4; 6.5)
Maternal hypertension or diabetes during pregnancy	p = 0.73 *	p < 0.01 *	p = 0.68 *	p = 0.76 *
No	29.9	90.6 (90.3; 91.0)	582.3 (581.6; 582.9)	6.42 (6.4; 6.5)
Yes	31.0	93.7 (92.1; 95.3)	582.9 (580.3; 585.4)	6.46 (6.2; 6.7)
Maternal age (years)	p < 0.01 **	p = 0.07 **	p = 0.45 **	p = 0.73 *
< 20	23.6	91.6 (90.6; 92.5)	582.4 (580.8; 584.1)	6.46 (6.3; 6.6)
20-29	29.4	90.8 (90.3; 91.2)	582.5 (581.6; 583.4)	6.41 (6.3; 6.5)
≥ 30	34.4	90.5 (89.8; 91.1)	581.8 (580.5; 583.0)	6.44 (6.3; 6.5)
Gestational age (weeks)	p = 0.22 *	p = 0.13 *	p = 0.88 **	p = 0.05 *
< 37	26.6	90.6 (89.6; 91.7)	582.0 (579.8; 584.3)	6.25 (6.1; 6.4)
37-39	31.3	91.3 (90.7; 91.9)	582.2 (581.1; 583.3)	6.49 (6.4; 6.6)
≥ 40	30.7	90.5 (90.0; 91.0)	582.2 (581.3; 583.1)	6.42 (6.3; 6.5)
Type of delivery	p < 0.01 *	p = 0.73 *	p = 0.94 *	p = 0.39 *
Vaginal	31.5	90.8 (90.4; 91.2)	582.3 (581.5; 583.1)	6.41 (6.3; 6.5)
Cesarean	25.8	90.9 (90.3; 91.6)	582.3 (581.2; 583.5)	6.47 (6.4; 6.6)
Birthweight (g)	p < 0.01 **	p = 0.63 **	p = 0.03 **	p = 0.57 **
< 2,500	22.5	90.8 (89.5; 92.2)	581.7 (578.7; 584.7)	6.36 (6.2; 6.5)
2,500-2,999	26.5	90.8 (90.1; 91.5)	581.4 (580.1; 582.8)	6.43 (6.3; 6.5)
3,000-3,499	30.8	90.6 (90.1; 91.2)	582.0 (580.9; 583.0)	6.42 (6.3; 6.5)
≥ 3,500	33.2	91.1 (90.5; 91.7)	583.5 (582.3; 584.6)	6.44 (6.3; 6.5)

95%CI: 95% confidence interval; BMI: body mass index.

Note: the definition of family income in tertiles is provided in the Supplementary Material (https://cadernos.ensp.fiocruz.br/static//arquivo/supl-e00044224_6433.pdf).

* Test for linear trend;

* Test for heterogeneity.

**Table 4:** Glycemia and lipid profile at 30 years according to confounding variables (n = 3,530). 1982 Pelotas (Brazil) birth cohort, 2012.

Variables	Glycemia (mg/dL)	Cholesterol (mg/dL)	HDL (mg/dL)	Non-HDL (mg/dL)
Mean (95%CI)	Mean (95%CI)	Mean (95%CI)	Mean (95%CI)
Sex	p < 0.01 *	p < 0.01 *	p < 0.01 *	p < 0.01 *
Male	92.5 (91.0; 94.0)	193.1 (191.1; 195.0)	53.8 (53.2; 54.3)	139.3 (137.4; 141.2)
Female	86.6 (85.6; 87.6)	189.5 (187.8; 191.2)	63.4 (62.8; 64.1)	126.1 (124.5; 127.6)
Family income at birth	p = 0.47 *	p < 0.01 **	p < 0.01 **	p = 0.15 **
1st tertile	89.6 (88.0; 91.2)	188.7 (186.4; 191.0)	57.6 (56.8; 58.4)	131.1 (128.8; 133.3)
2nd tertile	90.1 (88.7; 91.5)	190.8 (188.8; 192.9)	57.6 (56.9; 58.4)	133.2 (131.2; 135.2)
3rd tertile	88.8 (87.2; 90.4)	194.1 (191.9; 196.4)	60.8 (59.9; 61.6)	133.4 (131.2; 135.5)
Maternal BMI before pregnancy	p = 0.84 *	p = 0.83 **	p = 0.17 **	p = 0.45 **
Underweight	87.8 (85.6; 90.1)	190.7 (185.9; 195.5)	58.6 (56.8; 60.4)	132.1 (127.5; 1306.7)
Normal	89.2 (88.0; 90.4)	192.0 (190.3; 193.6)	59.1 (58.5; 59.7)	132.9 (131.3; 134.5)
Overweight	89.4 (87.6; 91.2)	191.9 (188.6; 195.2)	58.5 (57.4; 59.7)	133.4 (130.2; 136.6)
Obese	88.1 (85.6; 90.6)	191.9 (186.3; 197.5)	57.0 (54.7; 59.2)	135.0 (129.7; 140.2)
Maternal smoking	p = 0.03 *	p = 0.74 *	p = 0.15 *	p = 0.38 *
No	88.8 (87.8; 89.8)	191.1 (189.5; 192.7)	58.9 (58.3; 59.5)	132.2 (130.7; 133.7)
Yes	90.8 (89.2; 92.5)	191.6 (189.3; 193.8)	58.2 (57.4; 59.0)	133.3 (131.2; 135.5)
Maternal hypertension or diabetes during pregnancy	p = 0.09 *	p = 0.21 *	p = 0.38 *	p = 0.33 *
No	89.3 (88.4; 90.2)	191.1 (189.8; 192.3)	58.6 (58.1; 59.1)	132.4 (131.2; 133.7)
Yes	92.6 (87.3; 97.8)	194.6 (187.5; 201.7)	59.5 (57.6; 61.5)	135.1 (128.1; 142.1)
Maternal age (years)	p = 0.12 *	p = 0.16 *	p = 0.02 **	p = 0.12 *
< 20	91.7 (88.6; 94.8)	193.1 (189.8; 196.5)	57.7 (56.6; 58.9)	135.4 (132.2; 138.7)
20-29	89.0 (88.0; 90.0)	190.2 (188.6; 191.8)	58.5 (57.9; 59.1)	131.7 (130.1; 133.2)
≥ 30	89.4 (87.7; 91.1)	192.4 (189.8; 195.1)	59.5 (58.6; 60.4)	132.9 (130.4; 135.4)
Gestational age (weeks)	p = 0.80 *	p = 0.69 **	p = 0.25 **	p = 0.39 **
< 37	89.3 (85.8; 92.9)	190.7 (185.8; 195.6)	59.3 (57.9; 60.7)	131.3 (126.6; 136.1)
37-39	88.6 (87.3; 89.9)	190.9 (188.8; 193.1)	59.0 (58.1; 59.9)	131.9 (129.8; 134.1)
≥ 40	89.2 (88.0; 90.3)	191.4 (189.5; 193.4)	58.5 (57.8; 59.2)	132.9 (131.1; 134.8)
Type of delivery	p = 0.57 *	p = 0.50 *	p = 0.76 *	p = 0.56 *
Vaginal	89.7 (88.6; 90.7)	191.0 (189.5; 192.5)	58.6 (58.1; 59.2)	132.4 (130.9; 133.8)
Cesarean	89.1 (87.5; 90.7)	192.0 (189.4; 194.5)	58.8 (57.9; 59.7)	133.2 (130.7; 135.6)
Birthweight (g)	p = 0.12 *	p = 0.02 *	p < 0.01 *	p = 0.10 *
< 2,500	87.1 (85.3; 89.0)	185.7 (181.6; 189.7)	57.3 (55.7; 58.9)	128.3 (124.4; 132.2)
2,500-2,999	91.1 (88.8; 93.4)	193.7 (190.8; 196.6)	59.0 (58.0; 59.9)	134.7 (131.9; 137.6)
3,000-3,499	88.8 (87.6; 90.1)	191.8 (189.8; 193.9)	59.6 (58.8; 60.4)	132.2 (130.3; 134.2)
≥ 3,500	89.7 (88.1; 91.2)	190.0 (187.9;192.2)	57.7 (56.9; 58.5)	132.3 (130.2; 134.5)

95%CI: 95% confidence interval; BMI: body mass index.

Note: the definition of family income in tertiles is provided in the Supplementary Material (https://cadernos.ensp.fiocruz.br/static//arquivo/supl-e00044224_6433.pdf).

* Test for heterogeneity;

** Test for linear trend.

**Table 5:** Blood pressure, carotid intima-media thickness, and pulse wave velocity at 30 years according to breastfeeding duration (n = 3,530). 1982 Pelotas (Brazil) birth cohort, 2012.

Variables	n	Regression β (95%CI)					
		Mean arterial pressure (mmHg)		Carotid intima-media thickness (µm)		Pulse wave velocity (m/s)	
		Crude	Adjusted	Crude	Adjusted	Crude	Adjusted
Breastfeeding (months)		p = 0.50 *	p = 0.11 *	p = 0.55 **	p = 0.36 **	p = 0.39 **	p = 0.23 **
< 1	744	Reference (0)	Reference (0)	Reference (0)	Reference (0)	Reference (0)	Reference (0)
1 to < 3	908	-0.10 (-1.10; 0.90)	0.20 (-0.80; 1.10)	0.20 (-1.80; 2.10)	0.30 (-1.60; 2.20)	-0.11 (-0.30; 0.10)	0.13 (-0.30; 0.10)
3 to < 6	819	-0.30 (-1.30; 0.80)	0.10 (-0.90; 1.10)	0.20 (-1.80; 2.10)	0.70 (-1.20; 2.70)	-0.07 (-0.20; 0.10)	-0.08 (-0.20; 0.10)
6 to < 12	473	0.60 (-0.60; 1.80)	0.80 (-0.30; 2.00)	1.50 (-0.80; 3.80)	2.00 (-0.30; 4.30)	0.04 (-0.10; 0.20)	0.06 (-0.10; 0.30)
≥ 12	584	0.20 (-1.00; 1.30)	0.70 (-0.30; 1.80)	-0.60 (-2.70; 1.60)	-0.40 (-2.60; 1.80)	0.01 (-0.20; 0.20)	0.01 (-0.20; 0.20)
Predominant breastfeeding (months)		p = 0.77 *	p = 0.19 *	p = 0.35 **	p = 0.13 **	p = 0.41 **	p = 0.37 **
< 1	906	Reference (0)	Reference (0)	Reference (0)	Reference (0)	Reference (0)	Reference (0)
1 to < 2	467	-0.40 (-1.60; 0.80)	-0.30 (-1.40; 0.80)	1.30 (-0.90; 3.50)	1.50 (-0.70; 3.70)	-0.09 (-0.30; 0.10)	-0.10 (-0.30; 0.10)
2 to < 3	696	-0.10 (-1.10; 0.90)	0.60 (-0.30; 1.60)	1.40 (-0.60; 3.40)	2.10 (0.10; 4.00)	-0.12 (-0.30; 0.10)	-0.11 (-0.30; 0.10)
3 to < 4	921	-0.50 (-1.40; 0.50)	0.20 (-0.70; 1.10)	1.10 (-0.80; 2.90)	1.70 (-0.10; 3.60)	0.01 (-0.10; 0.20)	0.01 (-0.10; 0.20)
≥ 4	427	0.10 (-1.10; 1.30)	0.70 (-0.40; 1.90)	-0.50 (-2.80; 1.80)	-0.10 (-2.40; 2.20)	0.03 (-0.20; 0.20)	0.04 (-0.20; 0.20)

95%CI: 95% confidence interval.

Note: adjusted for genomic ancestry, sex, family income and maternal schooling at birth, asset index at childhood, maternal smoking, maternal hypertension and diabetes during pregnancy, maternal age, mode of delivery, maternal body mass index (BMI) before pregnancy, gestational age, and birthweight. Mean arterial pressure also adjusted for use of oral contraceptives and height at 30 years old.

* Test for linear trend;

** Test for heterogeneity.

**Table 6:** Glycemia and lipid profile at 30 years according to breastfeeding duration (n = 3,530). 1982 Pelotas (Brazil) birth cohort, 2012.

Variables	n	Regression β (95%CI)							
		Glycemia (mg/dL)		Cholesterol (mg/dL)		HDL (mg/dL)		Non-HDL (mg/dL)	
		Crude	Adjusted	Crude	Adjusted	Crude	Adjusted	Crude	Adjusted
Breastfeeding (months)		p = 0.35 *	p = 0.69 *	p = 0.15 **	p = 0.22 **	p = 0.10 **	p = 0.25 **	p = 0.12 *	p = 0.26 *
< 1	744	Reference (0)	Reference (0)	Reference (0)	Reference (0)	Reference (0)	Reference (0)	Reference (0)	Reference (0)
1 to < 3	908	-1.5 (-4.1; 1.1)	-1.4 (-3.9; 1.2)	-4.0 (-7.7; -0.2)	-3.8 (-7.5; -0.1)	-0.7 (-2.1; 0.7)	-1.0 (-2.3; 0.2)	-3.3 (-6.9; 0.3)	-2.8 (-6.3; 0.8)
3 to < 6	819	-1.9 (-4.6; 0.7)	-1.5 (-4.1; 1.2)	-0.3 (-4.1; 3.5)	-0.6 (-4.4; 3.3)	0.8 (-0.6; 2.1)	0.1 (-1.2; 1.4)	-1.0 (-4.7; 2.7)	-0.6 (-4.3; 3.0)
6 to < 12	473	-1.6 (-4.7; 1.4)	-1.3 (-4.3; 1.8)	-2.7 (-7.2; 1.7)	-3.4 (-7.9; 1.1)	1.3 (-0.3; 2.9)	0.5 (-1.0; 2.0)	-4.0 (-8.3; 0.3)	-3.9 (-8.2; 0.4)
≥ 12	584	-1.4 (-4.3; 1.5)	-0.6 (-3.6; 2.3)	-3.2 (-7.4; 1.0)	-2.6 (-6.8; 1.7)	0.2 (-1.3; 1.8)	-0.3 (-1.7; 1.2)	-3.5 (-7.5; 0.6)	-2.3 (-6.3; 1.7)
Predominant breastfeeding (months)		p = 0.17 *	p = 0.47 *	p = 0.36 **	p = 0.42 **	p = 0.03 *	p = 0.33 **	p = 0.12 *	p = 0.41 *
< 1	906	Reference (0)	Reference (0)	Reference (0)	Reference (0)	Reference (0)	Reference (0)	Reference (0)	Reference (0)
1 to < 2	467	-0.8 (-3.8; 2.2)	-0.9 (-3.8; 2.1)	-4.4 (-8.7; -0.1)	-4.3 (-8.6; 0.1)	-0.7 (-2.2; 0.9)	-0.7 (-2.2; 0.8)	-3.7 (-7.9; 0.4)	-3.6 (-7.7; 0.6)
2 to < 3	696	-2.1 (-4.7; 0.5)	-1.4 (-4.0; 1.2)	-2.1 (-5.9; 1.7)	-2.0 (-5.8; 1.9)	0.1 (-1.3; 1.5)	-1.0 (-2.3; 0.3)	-2.2 (-5.9; 1.5)	-1.0 (-4.7; 2.7)
3 to < 4	921	-1.0 (-3.5; 1.4)	-0.2 (-2.6; 2.3)	-1.4 (-5.0; 2.2)	-1.5 (-5.1; 2.1)	1.3 (-0.1; 2.6)	0.2 (-1.0; 1.4)	-2.7 (-6.1; 0.7)	-1.7 (-5.1; 1.7)
≥ 4	427	-2.2 (-5.2; 0.9)	-1.7 (-4.8; 1.4)	-2.5 (-7.0; 2.0)	-2.0 (-6.5; 2.6)	0.9 (-0.7; 2.6)	0.3 (-1.3; 1.8)	-3.5 (-7.8; 0.9)	-2.2 (-6.5; 2.1)

95%CI: 95% confidence interval.

Note: adjusted for genomic ancestry, sex, family income and maternal schooling at birth, asset index at childhood, maternal smoking, maternal hypertension and diabetes during pregnancy, maternal age, type of delivery, maternal body mass index (BMI) before pregnancy, gestational age, and birthweight. Glycemia also adjusted for time since last meal.

* Test for linear trend;

** Test for heterogeneity.

## Discussion

In this population-based birth cohort, duration of total and predominant breastfeeding was not associated to metabolic cardiovascular risk factors (mean arterial pressure, carotid intima-media thickness, pulse wave velocity, glycemia, and blood lipids) at 30 years.

Meta-analyses of observational and randomized studies have shown that breastfed participants had lower systolic blood pressure ^11^ and were less likely to have type 2 diabetes ^8^ ^,^ ^11^, whereas total cholesterol was not associated with breastfeeding ^11^. Most studies on the long-term consequence of breastfeeding were observational and conducted in high-income countries, a scenario in which high socioeconomic status is associated with longer duration of breastfeeding ^6^, and residual confounding could have overestimated the magnitude of the associations.

Indeed, findings from the *Promotion of Breastfeeding Intervention Trial* (PROBIT) ^20^ - in which exchangeability between the comparison groups is expected - indicated that allocation to breastfeeding promotion group was not associated to blood pressure and BMI at a mean age of 6.5 years. The null result of the mentioned study could be considered as an indication that the association of breastfeeding with BMI and blood pressure is not causal. Still, compliance to the intervention group was low. Among the intervention group, 49.8% of the children were breastfed in the first six months of life, whereas this proportion was 36.1% among the control. Because noncompliance to allocated group tends to decrease the statistical power of analyses, the null results of the mentioned study should not be considered as proof of absence of association.

A potential mechanism linking breastfeeding to cardiovascular risk factors involves its association with obesity, which is mediated by breastfeeding effect on the infant gut microbiota ^21^, promotion of healthier dietary preferences ^22^, and influence on satiety regulation ^23^. A previous study conducted in the same population showed that breastfeeding moderated the association between the fat mass and obesity-associated genotype variant *rs9939609* and adiposity measures, being inversely associated with visceral fat thickness ^10^. Therefore, regarding the relation between visceral fat and metabolic measures, we expected breastfeeding could be a protective factor for the cardiometabolic outcomes assessed, but the great increase of overweight and obesity prevalence may have mitigated the protective effect ^24^.

This study has two important strengths: we were able to evaluate the long-term association of breastfeeding with several cardiometabolic factors in a large population-based cohort with no clear social pattern of breastfeeding, and adjusted the analysis for different socioeconomic variables, reducing the possibility of residual confounding. Moreover, selection bias is unlikely due to the low attrition rate at 30 years, the nondifferential follow-up rates regarding breastfeeding duration, and the reasonably similar follow-up rates for baseline characteristics (Table 1). Information was prospectively collected by trained fieldworkers and the metabolic measures were assessed with high standard instruments, following standardized procedures, which minimize measurement bias. As a limitation, we were not able to separately evaluate individuals who were never breastfed. Because of the considerably low frequency of this category, it was grouped with < 1 month of breastfeeding. Furthermore, glycemia was evaluated using non-fasting blood glucose and it may have introduced a nondifferential misclassification even after adjusting for time since last meal.

Concisely, our study suggests no association between breastfeeding duration and cardiometabolic risk factors in adulthood. While no significant links were found with cardiovascular indicators at 30 years old, it is central to understand that breastfeeding plays an important role in reducing infant and child mortality. Additionally, its positive association with human capital development is noteworthy, as individuals who experience breastfeeding tend to show higher intelligence quotient scores, better school performance, and increased income in adulthood. Therefore, the diverse benefits derived from breastfeeding underscore the critical importance of promoting and supporting this practice. Our findings do not diminish the overall significance of breastfeeding; instead, they emphasize the need for a comprehensive understanding of the multifaceted advantages.
