# Supplementary material for: Breastfeeding and cardiometabolic risk factors in adulthood: results from the Pelotas (Brazil) birth cohort, 1982
Source: Cad Saude Publica. 2025 Feb 24;41(1):e00044224. doi: 10.1590/0102-311XEN044224 (PMC11863628; doi:10.1590/0102-311XEN044224)
Supplement: Supplementary file 1 [file 1678-4464-csp-41-01-EN044224-s.pdf]

**Supplementary Material**

This Supplementary Material provides the definition of the variable family income in tertiles, presented in Tables 1, 3, and 4.

Family income was collected at birth and categorized into five groups based on minimum wage units: ≤ 1, 1.1-3, 3.1-6, 6.1-10, and > 10. Due to the lack of continuous income data, a principal components analysis was conducted using delivery payment mode, mother's schooling, height, and skin color to derive family income tertiles. The first component generated a ranking score for individuals within each income category. Cut-off points were then established to form three nearly equal-sized groups, with minor variations due to ties in the scores [1].

1. Barros AJ, Victora CG, Horta BL, Gonçalves HD, Lima RC, Lynch J. Effects of socioeconomic change from birth to early adulthood on height and overweight. Int J Epidemiol 2006;35:1233-8.
